# Supplementary figures and images for: Genome-Wide Identification and Characterization of Auxin Response Factor (ARF) Gene Family Involved in Wood Formation and Response to Exogenous Hormone Treatment in Populus trichocarpa
Source: Int J Mol Sci. 2023 Jan 1;24(1):740. doi: 10.3390/ijms24010740 (PMC9820880; doi:10.3390/ijms24010740)

Motif 1

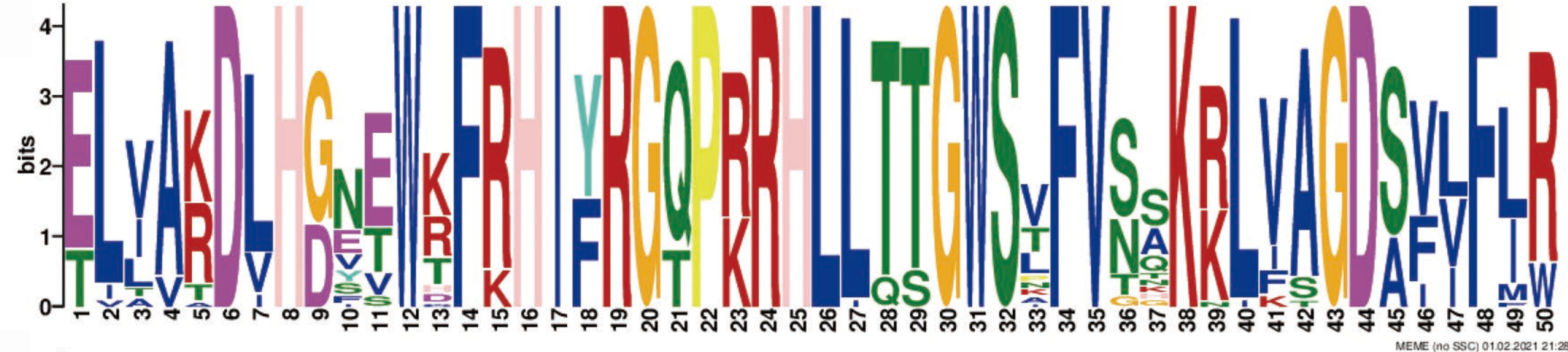

Motif 2

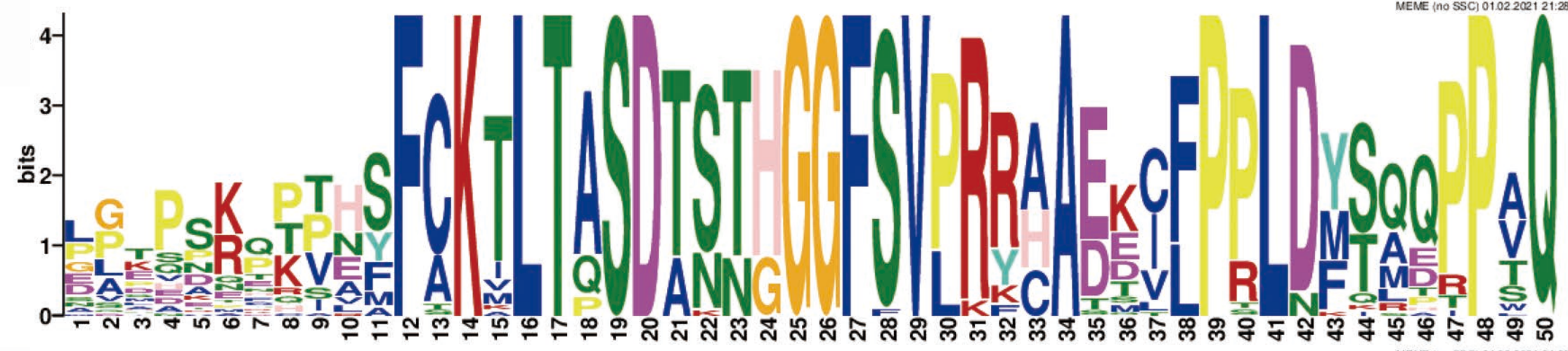

Motif 3

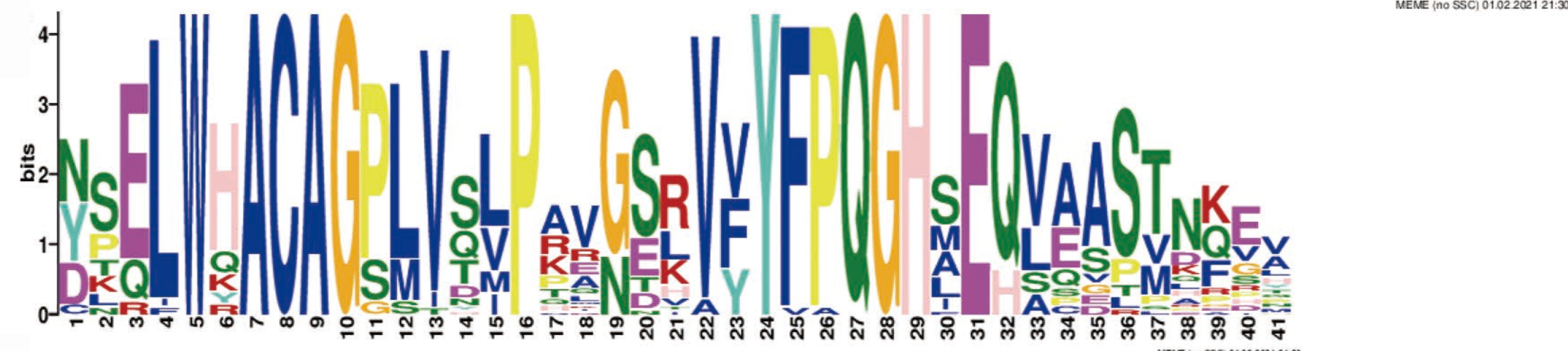

Motif 4

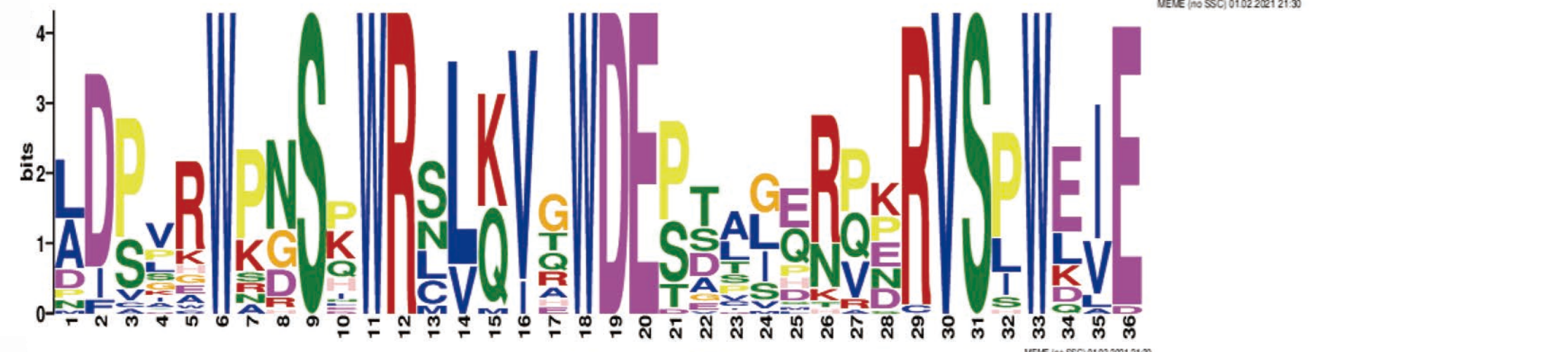

Motif 5

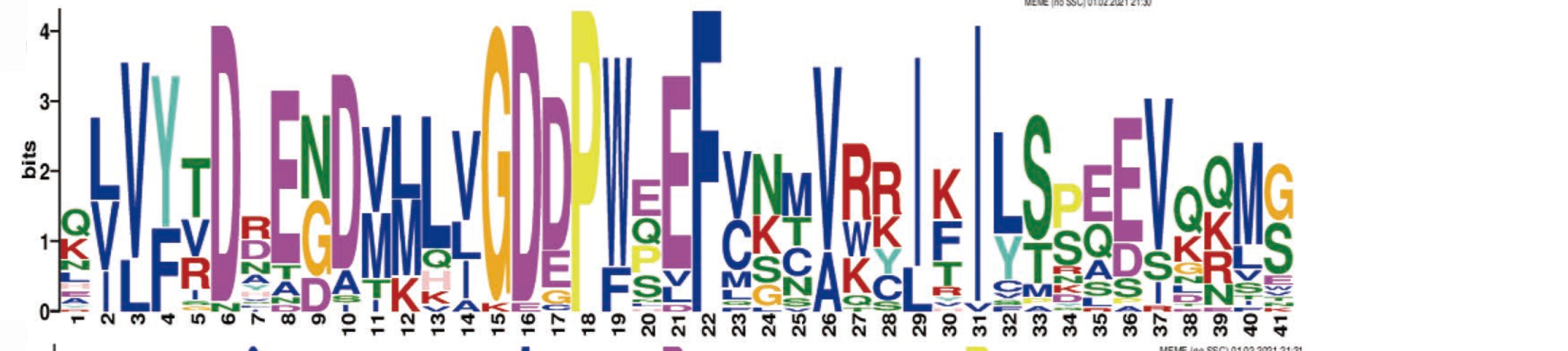

Motif 6

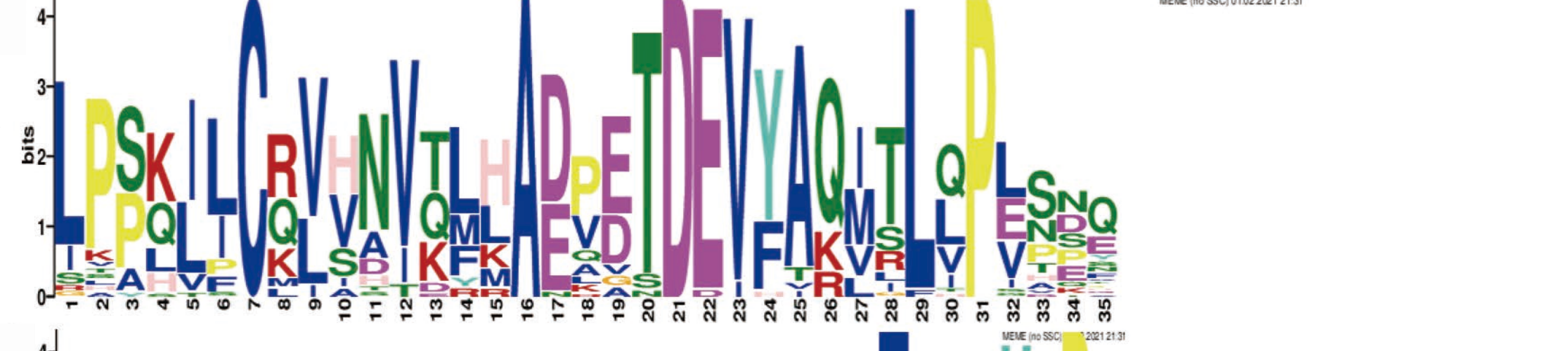

Motif 7

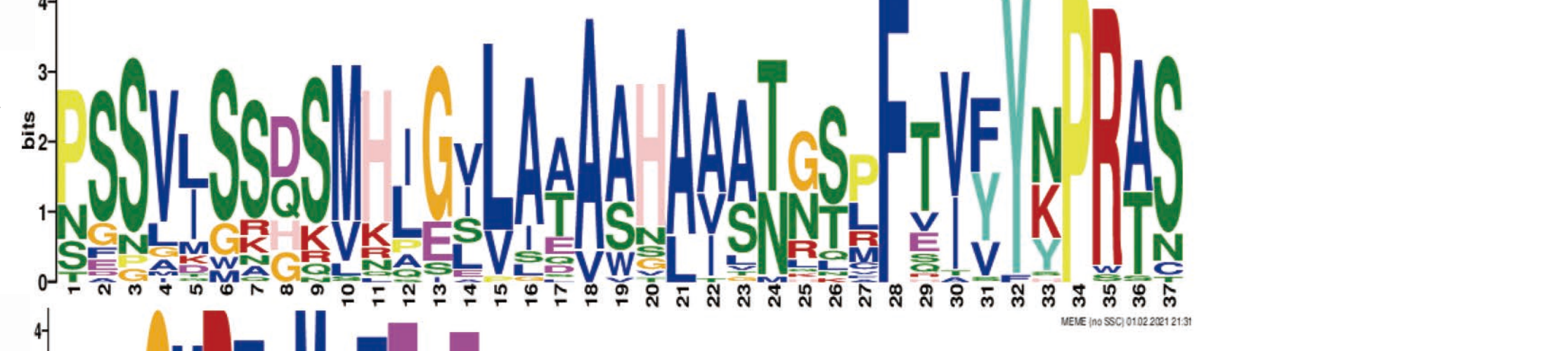

Motif 8

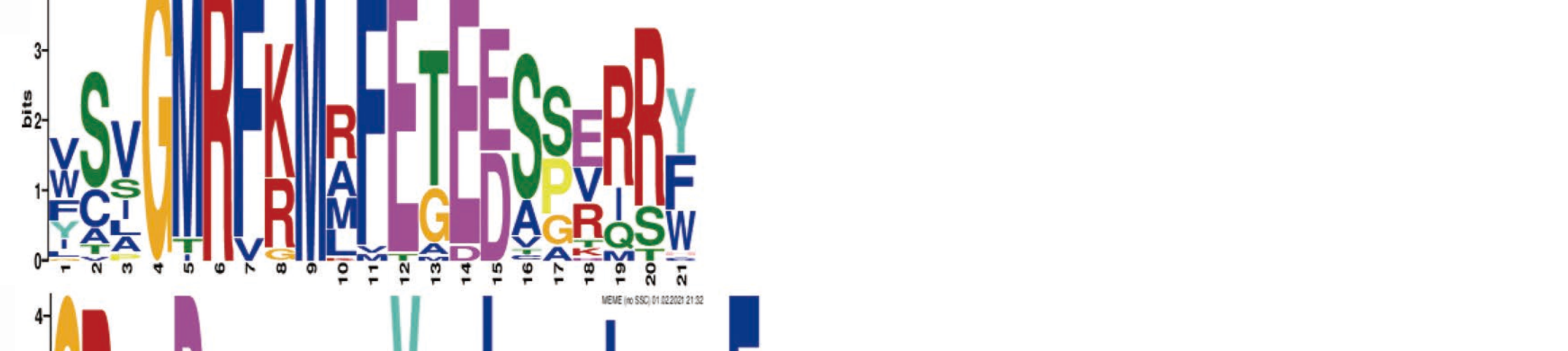

Motif 9

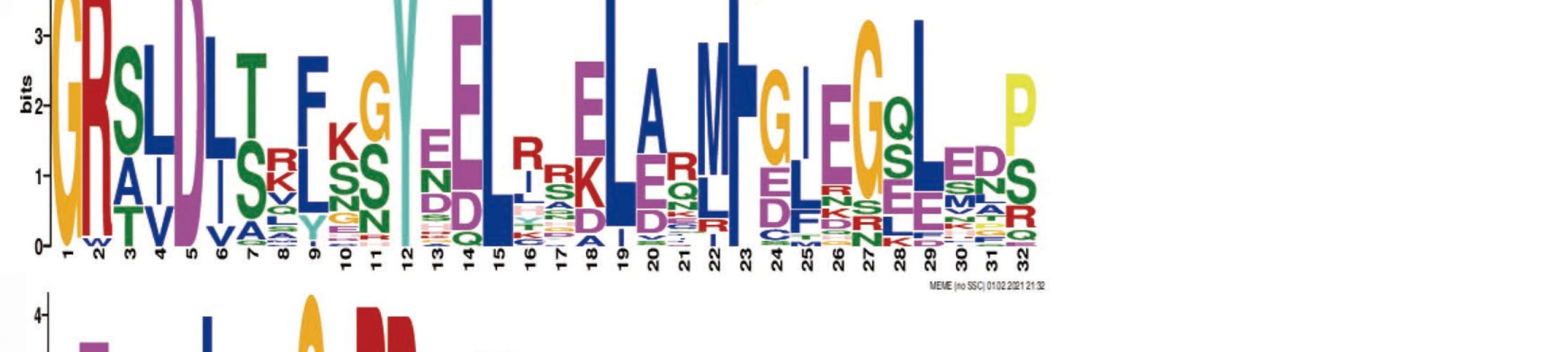

Motif 10

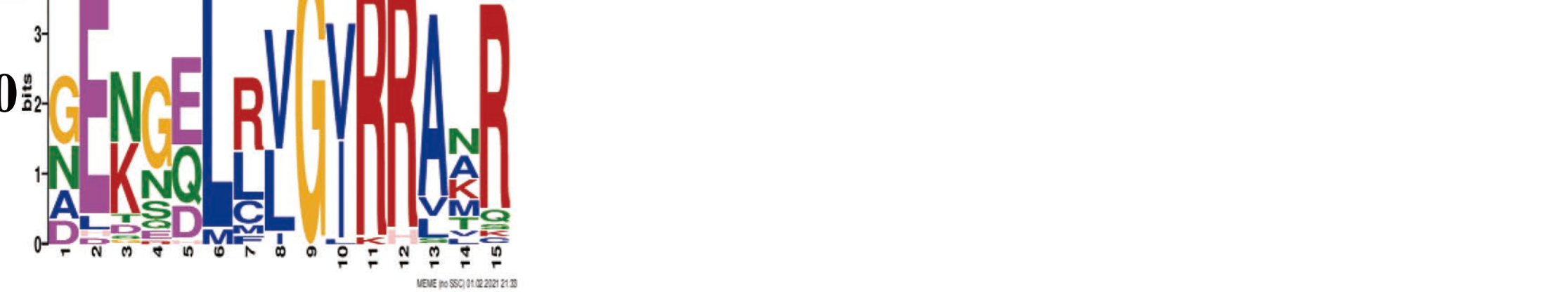

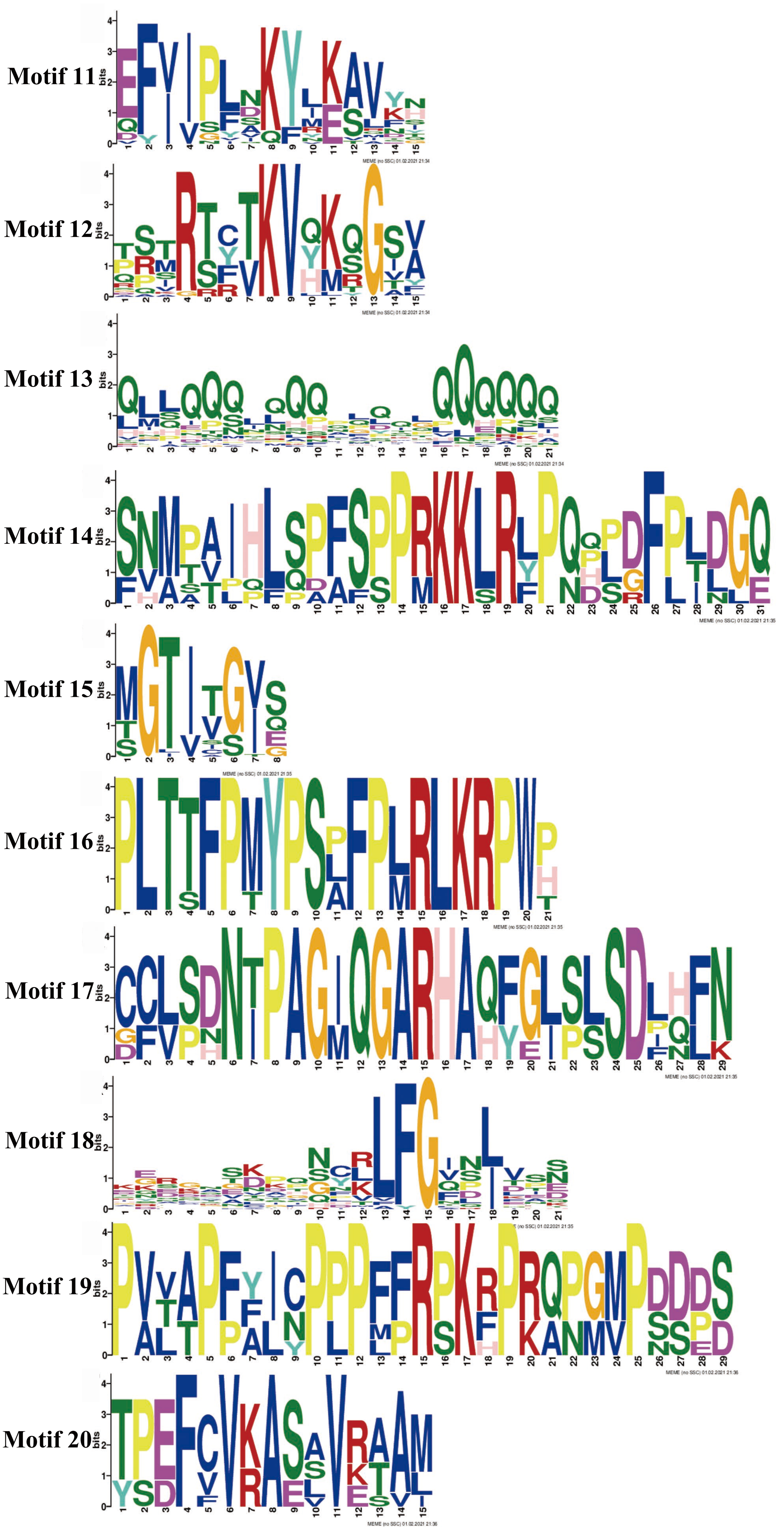

Supplement: Supplementary file 1 [file ijms-24-00740-s001.zip › Figure S1.pdf]

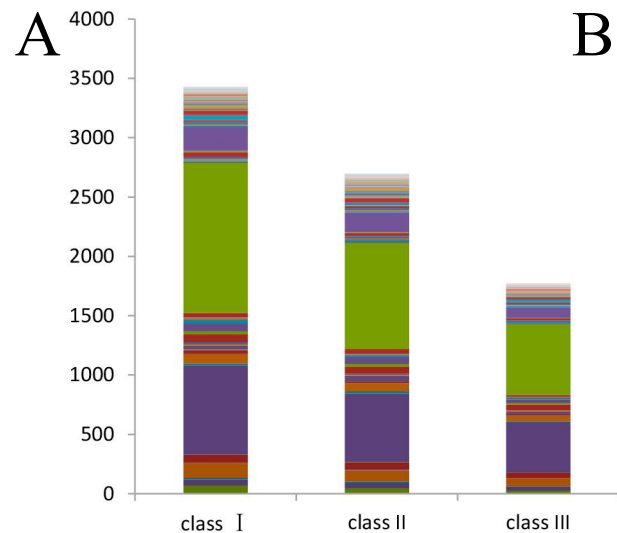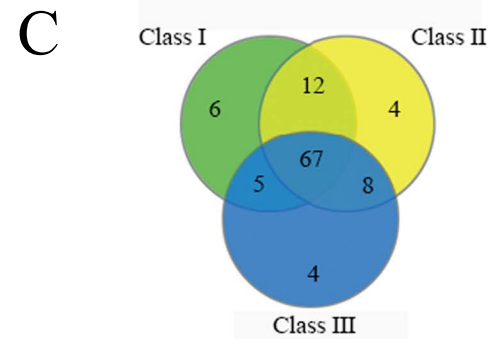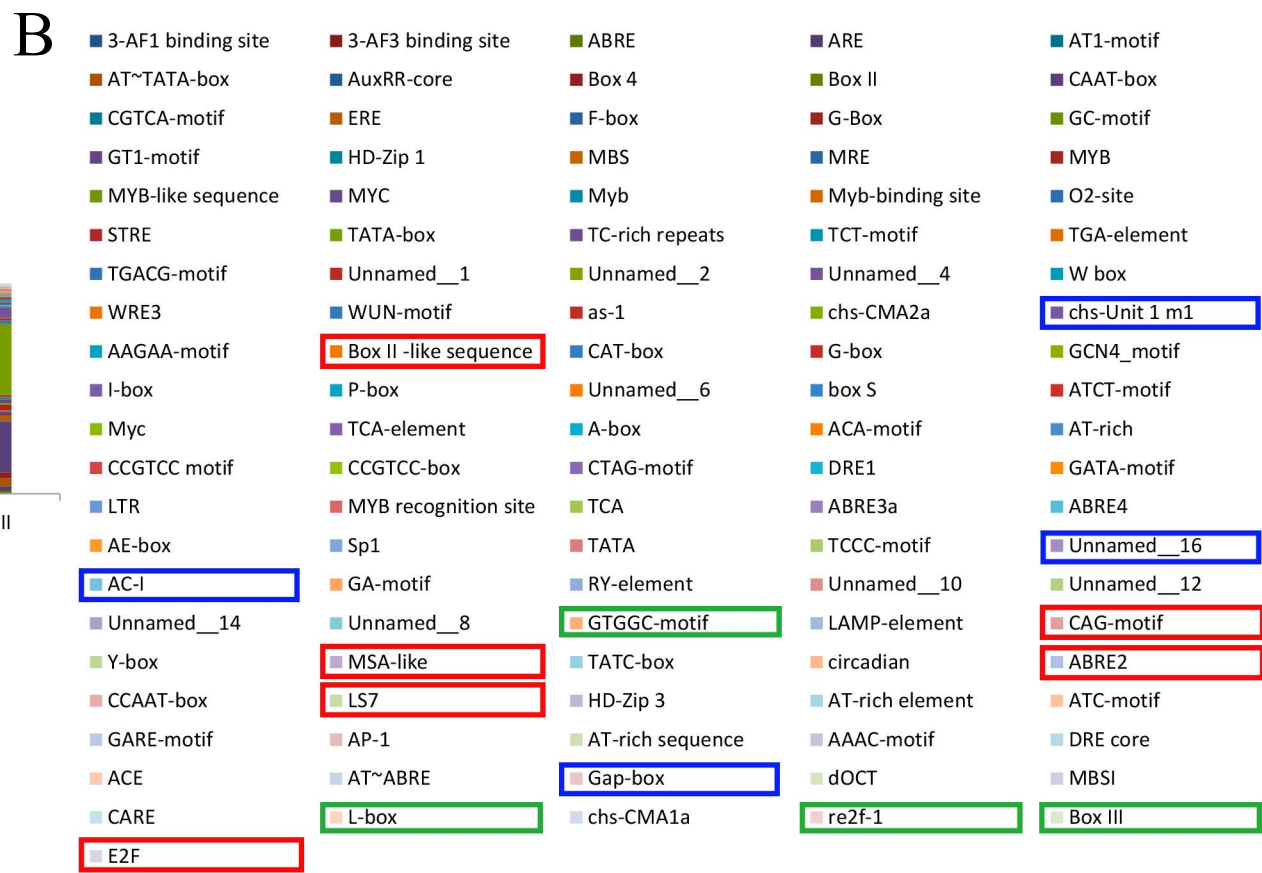

Supplement: Supplementary file 1 [file ijms-24-00740-s001.zip › Figure S2.pdf]
